# A Novel Use of Laryngoscope for Difficult Papanicolaou Smear Collection

**DOI:** 10.1155/2021/6986768

**Published:** 2021-09-23

**Authors:** Sarah Boudova, Caitlin Thomas, John Wolfe, Jeanne M. Schilder

**Affiliations:** ^1^Indiana University Department of Obstetrics and Gynecology, 550 University Boulevard, University Hospital 2440, Indianapolis, IN 46202, USA; ^2^Indiana University Department of Anesthesia, 1130 West Michigan Street Fesler Hall Room 204, Indianapolis, IN 46202, USA; ^3^Indiana University Department of Obstetrics and Gynecology, Division of Gynecologic Oncology, 550 University Boulevard, University Hospital 2440, Indianapolis, IN 46202, USA

## Abstract

The prevalence of cervical cancer has dropped significantly since introduction of the Papanicolaou (Pap) screen. The greatest risk factor for cervical cancer is inadequate screening. Altered pelvic anatomy can limit the ability to collect a Pap smear. In the presented case, a woman with a history of fibroids and bleeding presented for an exam under anesthesia. Traditional approaches for collecting a Pap smear failed. A GlideScope video laryngoscope was used to visualize the cervix, and a Pap smear was collected. The specimen was satisfactory, negative for intraepithelial lesion or malignancy, and HPV negative. A laryngoscope can be repurposed to visualize collection of a challenging Pap smear. Novel approaches for Pap smear collection and cervical cancer screening are needed and have the potential to save lives.

## 1. Introduction

Cervical cancer is the third most common gynecologic cancer in the US with an estimated 13,800 new cases and 4,290 deaths in 2020 [1]. However, these rates used to be significantly higher prior to the introduction of the Papanicolaou (Pap) test. Subsequently, the incidence of invasive cervical cancer has declined by over 50% [2]. Preinvasive lesions remain common [3], with 412,000 cases of cervical intraepithelial neoplasia diagnosed per year in the US [4], but screening allows these women to be treated prior to development of invasive cervical cancer.

Despite these advances, challenges remain. The greatest risk factor for cervical cancer is a lack of adequate screening [5–7]. Challenges in obtaining satisfactory Pap smears may stem from high body mass index, sharply anteverted/retroverted uterus, and altered anatomy due to surgery, fibroids, and other anatomic variants. Here, we present a case of a patient with significant distortion due to fibroids, whose Pap smear was collected using a laryngoscope for visualization.

## 2. Case

A 59-year-old nulliparous woman with a past medical history notable for HSV, premenopausal abnormal uterine bleeding, uterine fibroids with uterine fibroid embolization in the 2000s, now with postmenopausal bleeding, was referred to gynecologic oncology for management. Her gynecologist had attempted to collect a Pap smear and endometrial biopsy without success due to inability to adequately visualize her cervix. Her fibroids resulted in profound distortion of her pelvic anatomy, with her cervix tacked behind the pubic symphysis obstructing visualization and sample collection. Per the patient, this had also been the case with her previous gynecologist, and she was uncertain how long it had been since her last Pap smear. In addition to the referral, her gynecologist ordered a CA125 level and MRI. CA125 was within normal limits. The MRI showed a uterus measuring 20.4 × 14.1 × 18.5 cm with a distorted endometrial canal and extensive fibroids. The three largest fibroids were intramural and measured 11.2 × 9.1 × 11.4 cm, 7.8 × 5.9 × 6.6 cm, and 7.3 × 7.9 × 6.0 cm. There were also multiple subserosal fibroids. There was no hydronephrosis. The right adnexa appeared normal, and the left adnexa could not be visualized due to the fibroids. Lymph nodes appeared normal.

On presentation, the patient stated that her only symptoms recently had been abdominal pressure and postmenopausal bleeding that she described as intermittent spotting or blood with wiping. She went through menopause over one year earlier. She denied urinary complaints, dyspareunia, abdominal pain, distension, weight loss or gain, early satiety, constipation, or vaginal discharge. Past medical, family, and social history were noncontributory.

On exam, external genitalia appeared normal. No lesions or masses were palpated in the vagina, although blood was noted on glove after bimanual exam. On bimanual exam, the cervix was anterior behind the pubic symphysis. The uterus was midline, enlarged to 40-week size, nontender, fixed, and distorted with palpable masses. There were no palpable adnexal masses or lymph nodes. Rectovaginal exam was normal. On speculum exam, the cervix could not be visualized, and Pap smear and endometrial biopsy could not be performed in the office. It was discussed that her postmenopausal bleeding was likely secondary to her fibroids but that we could not rule out malignancy without a Pap smear and endometrial biopsy. The patient did not desire a hysterectomy if a benign process could be confirmed since her fibroids were relatively asymptomatic. The recommendation was made to proceed with an exam under anesthesia. She had a preoperative evaluation and was scheduled for the procedure.

In the operating room, general anesthesia was administered without difficulty. The patient was placed in dorsal lithotomy position. On bimanual exam, the uterus was enlarged to 40-week size. The cervix was barely palpable on bimanual exam despite manipulation including significant pressure from below as well as downward fundal pressure. The cervix could not be visualized with the Graves speculum or with a weighted speculum and right-angle retractor. A blind Pap was attempted under digital guidance; however, due to the length of the vaginal canal, curvature of the vaginal canal behind the pubic symphysis and the fact that the cervix was flush was the vaginal wall, it was unsuccessful. A rigid hysteroscope would have encountered the same challenges with positioning of the cervix behind the pubic symphysis preventing access. A flexible hysteroscope was not available. Discussing imaging tools that could achieve this level of curvature, it was noted that laryngoscopes are able to achieve this angle. In consultation with anesthesia, the decision was made to attempt visualization with a laryngoscope. Using a GlideScope video laryngoscope with a size 4 blade, the cervix was visualized posterior to the pubic symphysis. Blood was seen coming from the os. The os was flush with the vaginal wall. No gross cervical lesions were visualized. The vaginal walls were atrophic. A Pap spatula was taped to a rigid GlideScope intubating stylet, and the specimen was collected under direct visualization with the GlideScope. Similarly, an endocervical brushing was collected under GlideScope visualization after taping a brush to the end of a stylet. An attempt was made to collect an endometrial biopsy in a similar fashion with a Pipelle taped to a stylet and guided to the cervical os under visualization with the GlideScope; however, the Pipelle could not be advanced beyond approximately 1 cm secondary to inability to provide countertraction and the like presence of a fibroid. Scant bleeding was noted under visualization with the GlideScope. All instruments were removed from the vagina, and good hemostasis was noted. The patient tolerated the procedure well.

Pathology revealed fragments of benign squamous epithelium and rare endocervical glands. The Pap smear was satisfactory, negative for intraepithelial lesion or malignancy, and HPV negative.

The patient did well postoperatively, only requiring ibuprofen for cramping. She denied any vaginal pain. She continued to have a small amount of spotting. Given the inability to collect an adequate endometrial biopsy, she elected to proceed with hysterectomy. The pathology was benign.

## 3. Discussion

This report demonstrates a novel method of collecting a Pap smear in a patient with distorted pelvic anatomy. Repurposing a GlideScope video laryngoscope, we were able to visualize the cervix and rule out any gross evidence of invasive cervical cancer. We were also able to collect an adequate Pap smear under direct visualization. We further attempted an endometrial biopsy; however, the Pipelle could not be advanced beyond the cervix.

The Pap smear is a well-established method of screening for cervical cancer that has resulted in a remarkable decline in the incidence of cervical cancer since it has been widely adopted. Indeed, inadequate screening is the greatest risk factor for cervical cancer. Often, this lack of screening is due to social factors; however, biological factors can also play a role. Pap smears require visualization of the cervix, and unsatisfactory Pap smear rates are approximately 1%, although this is highly variable by site [8, 9]. The Bethesda System criteria require 8,000–12,000 or 5,000 well-preserved and well-visualized squamous cells for evaluation with conventional smears and liquid-based preparations, respectively [10]. Pap smears that do not meet these criteria are deemed unsatisfactory. These unsatisfactory Pap smears are at least as likely as satisfactory Pap smears to come from women subsequently diagnosed with CIN2 or more significant pathology [11–15]. Inability to visualize and properly sample the ectocervix contributes to the unsatisfactory samples. Women are advised to obtain a follow up Pap smear 2-4 months following an unsatisfactory result; however, practitioners will likely face the same challenges in obtaining an adequate specimen.

In this case, the patient had significantly distorted anatomy due to fibroids. It is estimated that 70% of white women and 80% of black women have fibroids by the age of 50 [16]. In addition to fibroids, surgery has the potential to alter pelvic anatomy. Roughly, one third of deliveries in the US occur via cesarean section [17]. Myomectomies, tubal ligations, salpingectomies, cystectomies, etc. also have the potential to cause adhesive disease and alteration of anatomy. Given the high prevalence of these conditions that could make the cervix inaccessible with a standard speculum exam, it is valuable to have alternative approaches. Traditional techniques include use of different size or style of speculum, repositioning the patient, exam under anesthesia, and bimanual exam to determine the location of the cervix with possible blind sample collection. However, repurposing other medical equipment as done in this case report can provide solutions to challenging cases. Of note, this was an off-label use of the video laryngoscope. It has not been FDA-approved for this purpose.

This case also raises the question of whether new instrumentation or diagnostic tests need to be developed for cervical cancer screening. Urine testing for HPV has been proposed and shows promise in research studies, but is not yet clinically available [18]. However, this method fails to visualize the cervix or provide cytology. We were able to obtain visualization and a Pap smear using a video laryngoscope, but if the patient had needed colposcopy, it is likely that this technique would not have been sufficient. The laryngoscope does not have the magnification or a blue-green light filter needed for colposcopy. It is also unlikely that any of our biopsy tools could have achieved the curvature needed to sample our patient's cervix. Modification of tools such as an endoscope or flexible hysteroscope could be beneficial for biopsy or even IUD placement in patients with altered anatomy. Flexible hysteroscopes are currently used for vaginoscopy in pediatric patients [19]. These tools could be repurposed for Pap smear, colposcopy, biopsies, or IUD placement. One such technology that has been developed is the Veda-scope which inflates the vagina with air and uses fiberoptics to visualize the cervix. This technology has been shown to achieve adequate cytology from women with typical pelvic anatomy [20]. Another technology, the POCkeT colposcope, uses a camera with a size of a tampon to visualize the cervix under white and green light and achieves results similar to visual inspection with traditional colposcopy for identifying precancerous lesions [21]. With the use of fiberoptics, a flexible version of this technology could be developed. Such technologies could also have use outside of the operating room and may even be less invasive and more acceptable to patients than traditional speculum exams.

The Pap smear is a life-saving screening tool. Currently, lack of access to Pap smears is a major risk factor for development of cervical cancer. Distorted pelvic anatomy due to fibroids or surgery can limit the ability to obtain a Pap smear using traditional methods. We successfully used a video laryngoscope for collection of a Pap smear. Developing new methods of screening for cervical cancer and obtaining the necessary specimens for Pap smears has the potential to save lives.

## Data Availability

The data are available in secure electronic medical record.
